# Physical Activity Before and During Pregnancy, Colorado Pregnancy Risk Assessment Monitoring System, 2012–2015

**DOI:** 10.5888/pcd17.190366

**Published:** 2020-07-09

**Authors:** Emily N. Ussery, Eric T. Hyde, Jennifer M. Bombard, Ashley L. Juhl, Shin Y. Kim, Susan A. Carlson

**Affiliations:** 1 Division of Nutrition, Physical Activity, and Obesity, National Center for Chronic Disease Prevention and Health Promotion, Centers for Disease Control and Prevention, Atlanta, Georgia; 2 Division of Reproductive Health, National Center for Chronic Disease Prevention and Health Promotion, Centers for Disease Control and Prevention, Atlanta, Georgia; 3 Health Statistics and Evaluation Branch, Center for Health and Environmental Data, Colorado Department of Public Health and Environment, Denver, Colorado; 4 Division of Congenital and Developmental Disorders, National Center on Birth Defects and Developmental Disabilities, Centers for Disease Control and Prevention, Atlanta, Georgia

## Abstract

We used 2012–2015 data from the Colorado Pregnancy Risk Assessment Monitoring System to describe changes in self-reported physical activity (PA) before and during pregnancy and used logistic regression to examine factors associated with regular PA. The prevalence of regular PA (ie, 30 or more minutes per day on 5 or more days per week) was 19.1% before pregnancy and decreased to 10.2% during pregnancy. At both times, adjusted odds of regular PA were lower among women who were overweight or had obesity before pregnancy than among those with normal weight. Findings suggest that most women with a recent live birth in Colorado, particularly those who are overweight or have obesity, are not obtaining many health benefits of PA either before or during pregnancy.

**Summary**

**What is already known about this topic?**

For most pregnant women, engaging in moderate-intensity physical activity has few risks but many health benefits. The *Physical Activity Guidelines for Americans,* 2nd edition, recommend that women with uncomplicated pregnancies who were physically active before pregnancy continue these activities during pregnancy.

**What is added by this report?**

Only 1 in 5 women in Colorado with a recent live birth reported engaging in regular physical activity during the 3 months before pregnancy, and the prevalence decreased to 1 in 10 during the last 3 months of pregnancy.

**What are the implications for public health practice?**

Health care providers can use evidence-based approaches to encourage women to maintain appropriate levels of physical activity before, during, and after pregnancy, with a focus on those who are overweight or have obesity before pregnancy.

## Objective

For most pregnant women, engaging in moderate-intensity physical activity (PA) has few risks but many health benefits, including reduced risk of gestational diabetes and postpartum depression (1). According to the *Physical Activity Guidelines for Americans,* 2nd edition, pregnant women should do 150 minutes or more of moderate-intensity aerobic PA per week, and women who routinely engaged in vigorous-intensity activities before pregnancy can continue those during pregnancy (1). Additionally, the American College of Obstetricians and Gynecologists (ACOG) recommends that pregnant women do 20 to 30 minutes of moderate-intensity exercise on most days of the week (2). However, population-based data describing PA levels of pregnant women and differences in PA before and during pregnancy are limited. A better understanding of factors associated with PA before and during pregnancy can help promote physical activity in this population. We examined differences in PA before and during pregnancy and correlates of regular PA among a population-based sample of women with a recent live birth in Colorado.

## Methods

The Pregnancy Risk Assessment Monitoring System (PRAMS) collects state-specific, population-based data on maternal behaviors and experiences before, during, and after pregnancy, and data are weighted to produce estimates representative of the state’s birth population (3). Women with a recent live birth in participating states are selected from birth certificate records by using stratified random sampling and mailed a survey 2 to 6 months after delivery. Women who do not respond by mail are next contacted by telephone. PRAMS methods have been approved by the Centers for Disease Control and Prevention’s institutional review board. We analyzed data from the Colorado PRAMS because it was the only state in phase 7 (2012–2015) that questioned participants (N = 6,180) about PA before and during pregnancy. Weighted response rates ranged from 59.1% (2014) to 67.4% (2012). Respondents were excluded if a health care provider advised them not to exercise (n = 549) or had missing data for PA (n = 131) or covariates (n = 139). The final sample was N = 5,361.

Participants reported the number of days per week they “participated in any physical activities or exercise for 30 minutes or more” during the 3 months before they got pregnant and during the last 3 months of pregnancy (response options, <1 day/week, 1–2 days/week, 3–4 days/week, ≥5 days/week, or told by a health care worker not to exercise). Respondent characteristics were obtained from the PRAMS questionnaire and children’s birth certificates, including age, race/ethnicity, education level, marital status, insurance at delivery, participation in the Special Supplemental Nutrition Program for Women, Infants, and Children (WIC), number of previous live births, preterm birth status, smoking, and prepregnancy body mass index (BMI, weight in kg divided by height in m^2^). WIC status was derived first from the survey and then from the birth certificate if missing on the survey to minimize missing data. For smoking status, a respondent was categorized as a smoker if reported on either data source to capture all smokers and minimize missing data.

We estimated the weighted prevalence of engaging in 30 or more minutes of PA on 5 or more days per week (“regular physical activity”) before and during pregnancy, overall, and by selected characteristics. This measure was selected to operationalize ACOG’s recommendation of 20 to 30 minutes of moderate-intensity exercise on most days of the week (2). We used logistic regression to estimate adjusted prevalence ratios of regular PA by respondent characteristics, while adjusting for other characteristics in the model. Results were considered significant at *P* < .05. Weighted analyses were performed in SUDAAN version 11.0 (Research Triangle Institute).

## Results

Most respondents were aged 25 to 34, non-Hispanic white, had greater than a high school diploma, had private insurance at delivery, and were normal weight before pregnancy (Table). During the 3 months before pregnancy, 19.1% were regularly active, and during the last 3 months of pregnancy, 10.2% were regularly active (Figure). Overall, 7.1% were regularly active before and during pregnancy. Less than half (45.3%) reported the same frequency of PA before and during pregnancy, 42.5% reported less frequency during pregnancy, and 12.3% reported higher frequency during pregnancy.

**Table:** Prevalence of Regular Physical Activity Before and During Pregnancy, by Selected Characteristics, Colorado PRAMS, 2012–2015

Characteristic	Sample Characteristics (N = 5,361)	Physical Activity Before Pregnancy^a^		Physical Activity During Pregnancy^b^	
	No.^c^ (%)^d^	%^e^ (95% CI)	APR^f^ (95% CI)	%^e^ (95% CI)	APR^f^ (95% CI)
**Age, y**					
≤24	1,294 (23.7)	18.4 (15.6–21.5)	1 [Reference]	12.4 (10.0–15.2)	1 [Reference]
25–34	3,088 (58.7)	18.3 (16.6–20.2)	1.00 (0.80–1.24)	9.5 (8.3–11.0)	0.91 (0.67–1.24)
≥35	979 (17.7)	22.4 (19.0–26.2)	1.29 (0.99–1.67)	9.6 (7.4–12.3)	0.98 (0.66–1.47)
**Race/ethnicity**					
Non-Hispanic white	3,617 (63.8)	22.0 (20.2–23.8)	1 [Reference]	10.8 (9.5–12.2)	1 [Reference]
Non-Hispanic black	151 (3.2)	11.3 (6.3–19.3)	0.57 (0.32–1.00)	8.6 (4.5–15.8)	0.77 (0.40–1.49)
Hispanic	1,283 (26.4)	13.2 (10.9–16.0)	0.72 (0.57–0.90)	9.2 (7.2–11.7)	0.83 (0.62–1.12)
Other	310 (6.6)	17.9 (13.1–23.9)	0.82 (0.61–1.12)	9.5 (5.8–15.1)	0.84 (0.52–1.36)
**Education level**					
<High school diploma	596 (12.0)	15.8 (12.0–20.5)	1 [Reference]	11.7 (8.6–15.9)	1 [Reference]
High school diploma	992 (18.7)	18.8 (15.7–22.2)	1.04 (0.77–1.42)	12.9 (10.2–16.2)	1.05 (0.71–1.56)
>High school diploma	3,773 (69.3)	19.7 (18.1–21.4)	0.85 (0.63–1.15)	9.2 (8.1–10.5)	0.69 (0.47–1.03)
**Marital status**					
Married	4,175 (79.2)	19.4 (17.9–21.0)	1 [Reference]	9.8 (8.7–11.1)	1 [Reference]
Other	1,186 (20.8)	17.9 (15.0–21.1)	0.98 (0.79–1.20)	11.7 (9.3–14.7)	0.99 (0.73–1.33)
**Insurance at delivery**					
Private	2,852 (55.2)	20.1 (18.3–22.1)	1 [Reference]	9.9 (8.6–11.5)	1 [Reference]
Medicaid	1,994 (36.1)	15.7 (13.6–18.0)	1.09 (0.88–1.35)	10.4 (8.6–12.4)	0.93 (0.67–1.27)
Other	515 (8.7)	26.5 (22.0–31.6)	1.42 (1.15–1.76)	11.6 (8.5–15.7)	1.07 (0.74–1.54)
**WIC during pregnancy**					
No	3,666 (70.2)	21.1 (19.4–22.9)	1 [Reference]	10.7 (8.8–13.0)	1 [Reference]
Yes	1,695 (29.8)	14.3 (12.2–16.7)	0.78 (0.63–0.97)	10.0 (8.8–11.4)	1.05 (0.78–1.43)
**Number of previous live births**					
0	2,385 (41.6)	22.1 (20.0–24.5)	1 [Reference]	11.9 (10.3–13.9)	1 [Reference]
1	1,644 (33.2)	17.8 (15.5–20.3)	0.81 (0.68–0.96)	9.0 (7.3–11.0)	0.79 (0.61–1.03)
≥2	1,332 (25.2)	15.6 (13.2–18.4)	0.75 (0.61–0.93)	9.1 (7.2–11.4)	0.81 (0.60–1.09)
**Preterm (<37 weeks) birth**					
No	4,169 (93.6)	19.2 (17.8–20.7)	1 [Reference]	10.4 (9.3–11.6)	1 [Reference]
Yes	1,192 (6.4)	17.3 (14.1–21.0)	0.95 (0.78–1.17)	8.1 (6.0–10.9)	0.78 (0.56–1.07)
**Smoked in the last 3 months of pregnancy**					
No	4,834 (92.1)	18.9 (17.5–20.4)	1 [Reference]	9.8 (8.8–11.0)	1 [Reference]
Yes	527 (7.9)	20.7 (15.9–26.4)	1.23 (0.95–1.59)	14.9 (10.8–20.1)	1.47 (1.03–2.09)
**Prepregnancy BMI status**					
Underweight	199 (3.3)	15.2 (9.5–23.4)	0.59 (0.37–0.95)	11.7 (6.4–20.3)	0.86 (0.47–1.58)
Normal weight	2,769 (52.8)	25.0 (22.9–27.2)	1 [Reference]	12.2 (10.7–13.9)	1 [Reference]
Overweight	1,170 (21.9)	12.8 (10.5–15.4)	0.55 (0.45–0.68)	7.6 (5.7–9.9)	0.63 (0.46–0.85)
Has obesity	763 (13.3)	10.7 (8.2–13.8)	0.47 (0.36–0.62)	6.7 (4.7–9.6)	0.52 (0.36–0.77)
Missing	460 (8.8)	13.4 (9.8–17.9)	0.59 (0.43–0.81)	9.7 (6.5–14.0)	0.75 (0.50–1.13)

Abbreviations: APR, adjusted prevalence ratio; BMI, body mass index; CI, confidence interval; PRAMS, Pregnancy Risk Assessment Monitoring System; WIC, Special Supplemental Nutrition Program for Women, Infants, and Children.

a Regular physical activity before pregnancy was defined as responding “5 or more days per week” to the question, “During the 3 months before you got pregnant with your new baby, how often did you participate in any physical activities or exercise for 30 minutes or more? For example, walking for exercise, swimming, cycling, dancing, or gardening.”

b Regular physical activity during pregnancy was defined as responding “5 or more days per week” to the question, “During the last 3 months of your most recent pregnancy, how often did you participate in any physical activities or exercise for 30 minutes or more?”

c Unweighted counts. From the initial sample of 6,180, respondents were excluded if they were advised not to exercise by a health care provider before or during pregnancy (n = 549) or were missing data on physical activity (n = 131) or covariates (n = 139). Compared with the analytic sample, respondents who were excluded due to missing data were more likely to be Hispanic, have lower education, be unmarried, be on Medicaid at delivery, and be on WIC during pregnancy. Variables were derived from the child’s birth certificate (age, race/ethnicity, education level, marital status, insurance at delivery, number of previous live births, and preterm birth), the self-reported PRAMS survey (prepregnancy BMI based on height and weight), or a combination of both data sources (WIC during pregnancy and smoking status during the last 3 months of pregnancy). WIC status was derived first from the survey and then from the birth certificate if missing on the survey to minimize missing data. For smoking status, respondents were categorized as smokers if they reported on either data source to capture all smokers and minimize missing data.

d Percentages weighted to be representative of women in Colorado with a recent live birth during the study period.

e Unadjusted percentage reporting regular physical activity at each time point.

f Models adjusted for all variables shown in the table.

**Figure:**
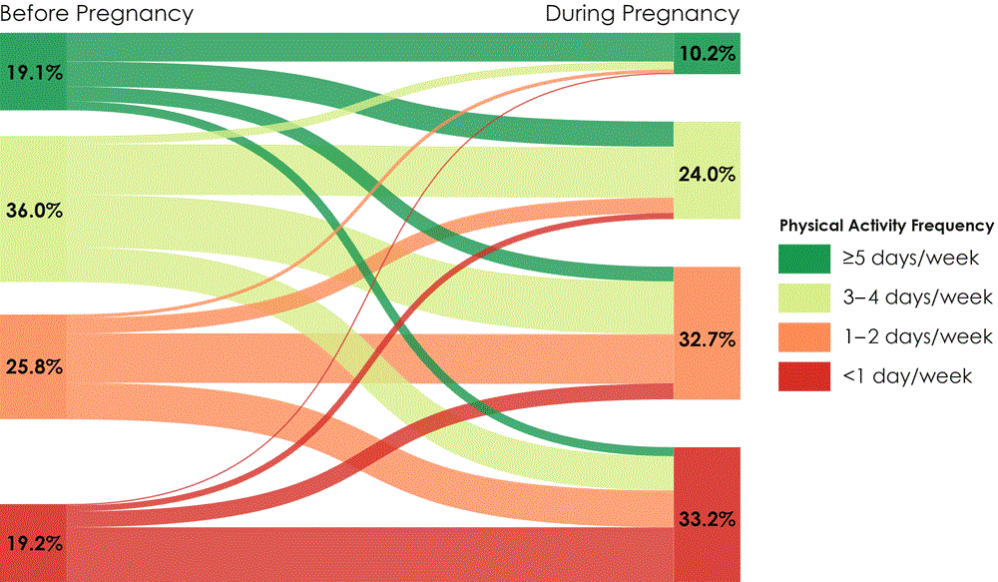
Changes in frequency of at least 30 minutes per day of physical activity before and during pregnancy among women with a recent live birth, Colorado Pregnancy Risk Assessment Monitoring System, 2012–2015.

**Table:** 

Physical Activity Frequency During Last 3 Months of Pregnancy, d/wk	%
**Women who exercised ≥5 d/wk 3 months before pregnancy**	19.1
≥5	7.1
3–4	6.2
1–2	3.6
<1	2.2
**Women who exercised 3 or 4 d/wk 3 months before pregnancy**	36.0
≥5	2.0
3–4	12.5
1–2	13.0
<1	8.5
**Women who exercised 1 or 2 d/wk 3 months before pregnancy**	25.8
≥5	0.8
3–4	3.8
1–2	12.2
<1	9.0
**Women who exercised <1 d/wk 3 months before pregnancy**	19.2
≥5	0.3
3–4	1.5
1–2	3.9
<1	13.5

In adjusted models, race/ethnicity, insurance at delivery, WIC during pregnancy, number of previous live births, and prepregnancy BMI status were associated with regular PA before pregnancy only (Table). Smoking status during the last 3 months of pregnancy was associated with regular PA during pregnancy only. Prepregnancy BMI status was the only characteristic significantly associated with regular PA both before and during pregnancy; women who were overweight or had obesity before pregnancy had lower odds of regular PA at both time points versus those with normal weight.

## Discussion

Approximately 1 in 5 women in Colorado with a recent live birth reported regular PA during the 3 months before pregnancy, and prevalence decreased to 10% during the last 3 months of pregnancy. Overall, 43% reported decreased PA, despite recommendations of maintaining prepregnancy PA levels during pregnancy (1). Findings suggest that most women with a recent live birth in Colorado, particularly those who are overweight or have obesity, are not obtaining many health benefits of PA before or during pregnancy.

Our findings are consistent with those of previous studies that indicated low PA levels during pregnancy (4,5). From 2007 through 2014, an estimated 12.7% to 45.0% of pregnant women met the ACOG recommendation, depending on how it was operationalized (4). Other studies show that PA overall decreases from prepregnancy to pregnancy, although population-based studies are limited (6–8). A 2008 PRAMS study found that 30 or more minutes per day of PA on 5 or more days per week was reported by 12.6% of women before pregnancy and by 9.3% during the last trimester (8). Women report several barriers to PA participation during pregnancy, including contradictory information about the risks and benefits of PA and lack of guidance from health care providers (9,10). Providers can follow ACOG recommendations to educate women on the pregnancy-related benefits of PA and encourage them to maintain appropriate PA levels before, during, and after pregnancy (2).

We found that the prevalence of regular PA before and during pregnancy was low across demographic groups, particularly among women who were overweight or had obesity before pregnancy. Previous studies report similar associations between PA durin­g pregnancy and BMI (6,8). Obesity is associated with several reproductive complications, and participation in regular PA improves weight status and the risk profile of pregnant women (11). Providers can use evidence-based approaches, such as motivational interviewing, to help patients who are overweight or have obesity incorporate more PA into their weekly routines (12).

Our study had several limitations, including self-reported and retrospective assessment of PA, assessment at one time point, and data from only one state. Moreover, the survey did not assess PA intensity or total duration, precluding estimates of adherence to the national guideline of 150 minutes or more per week of moderate-intensity PA (1). Regardless, our findings highlight the importance of promoting regular PA before pregnancy and maintaining regular PA levels throughout pregnancy.
